# “It’s different here” Afghan refugee maternal health experiences in the United States

**DOI:** 10.1186/s12884-024-06678-7

**Published:** 2024-07-16

**Authors:** Heidi J. Worabo, Fatima Safi, Sara L. Gill, Moshtagh Farokhi

**Affiliations:** 1 School of Nursing, University of Texas Health Science Center San Antonio, 7703 Floyd Curl Drive. Mail Code 7951, San Antonio, Texas 78229‑3900 USA; 2 HCA Medical City Consortium, 3301 Matlock Road, Arlington, Texas 76015 USA; 3 School of Dentistry, University of Texas Health Science Center San Antonio, 7703 Floyd Curl Drive. Mail Code 7914, San Antonio, Texas USA

**Keywords:** Maternal health, Refugees, Reproductive healthcare, Afghan women, Culture, Qualitative, Birthing experience

## Abstract

**Background:**

The number of Afghan families in the US has grown over the past two decades, yet there is a paucity of research focused on their maternal healthcare experiences. Afghan families have one of the highest fertility rates in the world and typically have large families. As the US faces rising maternal mortality rates, it is crucial to understand factors that affect health outcomes for culturally distinct groups. We aimed to better understand Afghan women’s maternal health experiences in South Texas as a step toward designing culturally sensitive care.

**Methods:**

Using a qualitative descriptive design, twenty Afghan women who gave birth in the US within the past 2 years participated in audio-recorded interviews. The first and second authors conducted each interview using a semi-structured interview guide. The authors used an in vivo coding method and qualitative content analysis of the transcribed narrative data.

**Results:**

We identified three broad categories with corresponding sub-categories: 1) Maternal Healthcare Experiences: pregnancy, birthing, and postpartum, 2) Communication: language barrier, relationship with husband, and health information seeking, 3) Access to Care: transportation and financing healthcare. The participants expressed perspectives of gratefulness and positive experiences, yet some described stories of poor birth outcomes that led to attitudes of mistrust and disappointment. Distinct cultural preferences were shared, providing invaluable insights for healthcare providers.

**Conclusions:**

The fact that the Afghan culture is strikingly different than the US mainstream culture can lead to stereotypical assumptions, poor communication, and poor health outcomes.

The voices of Afghan women should guide healthcare providers in delivering patient-centered, culturally sensitive maternity care that promotes healthy families and communities.

**Supplementary Information:**

The online version contains supplementary material available at 10.1186/s12884-024-06678-7.

## Background

The people of Afghanistan have seen one violent conflict after another for more than five decades. From the Soviet Union invasion in 1979 to the Afghan Civil War and then the United States’ (US) military invasion in 2001, there has been constant insecurity and economic hardship [1, 2]. Despite the controversial nature of the US 20-year military presence in Afghanistan, the country’s health system made strides toward improved health outcomes, including lowering the infant mortality rate from 66 to 45 deaths per 1000 live births and from 87 to 55 deaths per 1000 children under five years old [3]. However, with the US withdrawal from Afghanistan in 2021, there was an immediate takeover by the Taliban. Since then, the health system has deteriorated due to a lack of funding, natural disasters, and political unrest [4]. Afghan families have one of the highest fertility rates (4.53 children per woman) in the world [5] and have the eighth highest maternal mortality rate at 621 deaths per 100,000 [6]. The median age of marriage for Afghan women is 18.5 years, with 22.5% of married women using contraception [3]. As of 2021, the literacy rate for women was 23% [7]. Afghan women have faced compounding factors that threaten their health and wellbeing.

During the US exit from Afghanistan in August of 2021, 124,000 people were airlifted out of Afghanistan, of which 55,000 Afghans were placed in US military bases before resettling in cities across the country. Military medical personnel who were tasked with assessing and treating Afghans on military bases concluded that reproductive health must be prioritized [8].

Refugee women in the US arrive from diverse cultures, languages, and political situations; however, they share the common experience of fleeing their homes due to threats to their lives. Forced displacement results in vulnerability to poor maternal health outcomes, including preterm labor, spontaneous abortion, physical and sexual trauma, and infectious disease [9, 10]. For women of reproductive age, pregnancy poses certain risks to their health that can be exacerbated through the migration process [11]. Sharma et al. (2024) found that Afghan women migrating through Serbia faced discrimination, trauma, and unreliable reproductive health services [12].

Once refugees arrive for permanent resettlement in the US, they face healthcare access challenges, including healthcare navigation, language barriers, and complicated, fragmented funding sources for healthcare [13]. A study involving Arab refugee women residing in the US found acculturation stress and posttraumatic stress were correlated with increased depressive symptoms and impairment of maternal-infant bonding [14]. A scoping literature review of maternal health among resettled refugee women in the US found refugee women initiated prenatal care later and attended fewer prenatal visits compared to women born in the US. The researchers found an underrepresentation of certain ethnic groups in refugee maternal studies, including women from Afghanistan [15].

Maternal research in Australia with Afghan refugees found that health information and community engagement during and after pregnancy were essential as health literacy and language barriers affected their quality of care [16, 17]. An exploration of the emotional wellbeing of Afghan women during and after giving birth in Australia found that women faced a lack of social connectedness and feelings of isolation [18]. A study based in Iran revealed that Afghan refugee women had worse maternal health outcomes compared to the general population [19]. Interviews with Afghan women who had almost died from a maternal health complication in Iran cited health professionals’ discriminatory lack of attention and delays in diagnosing complications as causes of poor health outcomes [20]. Through qualitative interviews with Afghans in Iran, Dadras et al. (2020) found that lack of health insurance reduced access to prenatal care, and the high costs of hospital care burdened Afghan families [21].

Despite the rising number of Afghans living in the US [22], Afghan maternal health research is lacking. What are Afghan women’s maternal health experiences in a US context? As healthcare professionals in South Texas, US, we aimed to gain an understanding of their experiences of being pregnant, giving birth, and postpartum care in the US to guide the provision of maternity care.

## Methods

### Study design

We utilized qualitative descriptive methodology to explore Afghan women’s maternal health experiences in South Texas [23]. Qualitative descriptive methods are less interpretive than other qualitative methods such as phenomenology or grounded theory, allowing the researcher to present a thorough description of the phenomenon in everyday language. The method is commonly used in practice disciplines and does not necessarily rely on abstract or theoretical frameworks. This method was chosen because we wanted to stay close to the surface of the data (the participants’ descriptions). Qualitative descriptive studies provide a comprehensive summary of events with relevance to practitioners and policymakers [23].

### Study setting and participant recruitment

The study setting was a refugee social service agency located in a city in South Texas. In addition to several assistance programs, the agency operates a program for postpartum women. The first and second authors recruited participants who attended the postpartum program. Inclusion criteria were women aged 18 years or older, originally from Afghanistan, and who had given birth in the US within the past 2 years. The second author, who speaks Pashto, Dari, and English, used a script to call and request interviews. Agency staff provided phone numbers. Fifty-two women were contacted from the list; 17 could not be reached through the telephone number on file; 15 declined to participate. A convenience sample of 20 women agreed to participate. To ensure that the women were not pressured into participating, the list of women who declined or accepted was not shared with the agency staff. Participant information was stored in a password-protected file that only the second author could access. To decrease barriers to attending the interview (childcare responsibilities, lack of transportation, etc.), we offered the participant options for conducting the interview—in-person at their home or the social service agency, over Zoom, or by telephone. A $30 gift card was given to each participant after the interview.

### Data collection

Consistent with qualitative descriptive design, the data collection included moderately structured open-ended questions [23]. Prior to beginning the interview, the second author assisted each participant with filling out the Demographic Form (Appendix A). She interpreted the Form verbally and wrote their responses in English. The first and second authors conducted 20 interviews using a semi-structured interview guide (Appendix B). The first author led the interviews in English, and the second author interpreted between English and Pashto or Dari languages, depending on the participant’s preference. The interviews were audio-recorded, and a professional transcription company transcribed the English on the recordings verbatim. Following the reflexive and interactive nature of qualitative content analysis [24], we modified the interview guide after the first eight interviews by adding follow-up questions under each primary question (Appendix C). The first 8 participants were not re-interviewed with the new follow-up questions.

### Data analysis

The second author, fluent in Pashto and Dari, validated the English transcripts’ consistency with the audio recordings' non-English content. The authors carefully read the transcripts multiple times. The first and second authors reflected on each interview by comparing the notes they had made during the live interviews. Congruent with qualitative content analysis, the authors used in vivo coding [24]. The iterative process of line-by-line coding was manually completed by two of the authors. Then, they came together to discuss intercoder agreements between their list of codes. The four authors reviewed the list of codes and began to cluster the codes according to similarity. Through in-depth analysis and debriefing of the meaning of the data, the authors grouped the codes into broader categories with three sub-categories under each broad category [25]. Illustrative quotes were highlighted and listed under each of the sub-categories. The authors discussed reflexivity by acknowledging how author biases and experiences could shape the interpretation of the data [24, 25].

### Ethical considerations

The university’s institutional review board approved the study (Protocol #20210606NRR). Each participant provided audio-recorded verbal informed consent before beginning the interview. Due to the possibility of sharing sensitive information during the interviews, we ensured that women who had further questions about their health status, healthcare resources, and any emotionally charged topics were addressed immediately after the interview. Since the authors are healthcare professionals at a multispecialty academic health science center and involved in the city’s refugee assistance programs, we were able to address any concerns that arose and provide referrals.

## Results

The interviews lasted between 23 and 71 min, with an average time of 48 min. Six interviews were conducted in person, and 14 were over the phone with a 3-way conversation between the first author, the second author, and the participant. The details of their demographic data are displayed in Table 1.

**Table 1:** Demographics of the participants (*n* = 20)

	**n**	**%**
Country of origin: Afghanistan	20	100
Religion: Islam	20	100
Married	20	100
Primary language		
Pashto	15	75
Dari	5	25
Education level		
None	13	65
Elementary	3	15
Secondary	4	20
Can read or write:		
Pashto	3	15
Dari	2	10
English	3	15
English spoken proficiency		
None	11	55
Conversational	9	45
Fluent	0	0
Unemployed	19	95
Has health insurance	17	85
	**Mean**	**Range**
Age	38 years	23–40
Number of pregnancies	5	2–10
Number of children	4.1	2–7
Time in the U.S	5.1 years	1.5–11

The participants’ words painted common perspectives of gratefulness and positive experiences in the US, yet three of the women described stories of poor birth outcomes that led to attitudes of mistrust and disappointment. Distinct cultural preferences were shared, providing invaluable insights for healthcare providers. “It’s different here” was the common theme across participants’ accounts*.* The three broad categories that emerged from the data were 1) Maternal Healthcare Experiences, 2) Communication, and 3) Access to Care, each with sub-categories (Table 2).

**Table 2:** Categories

Category	Sub-Category	Codes
**Maternal Healthcare Experiences**	Pregnancy	Helpful Close monitoring Self-pregnancy Economic situation Nutrition
	Birthing	Doctors and nurses check on me Epidural made it easy Not comfortable with males in the room Want natural births
	Postpartum	Breastfeeding is smooth Space between children Didn’t get information
**Communication**	Language Barrier	We don’t know the language Prefer my husband to interpret Keep it to ourselves
	Relationship with Husband	I share with my husband first Husband was the biggest support Respect their husbands
	Women’s Information Seeking	Learn from female family members Shameful topic Busy with kids Responsibilities
**Access to Care**	Transportation	I can’t drive Husband has to drive me Cancel appointments
	Financing Healthcare	If I have insurance, I’ll go to the doctor Need Medicaid Need financial support

### Maternal healthcare experiences

This category encompasses the women’s experiences with maternity care in the US. Nineteen of the participants had also given birth to a child[ren] in Afghanistan and therefore, reflected on the differences between their experiences in the two countries. This category included prenatal care, how they took care of themselves during pregnancy, their labor and delivery experiences in the hospital setting, and their postpartum practices, preferences, and engagement with healthcare services.

#### Pregnancy

The women were pleased with the prenatal care they received in the US. They felt the providers were caring and helpful, and closely monitored them during pregnancy. They contrasted this with their experience of being pregnant in Afghanistan, where only two of the women had prenatal care. The women indicated not receiving special care and only sought healthcare if they had bleeding or pain. As one participant noted,

“*It was really self-pregnancy back home”* (Participant 12).

They identified the poor economic situation in Afghanistan as what prevented them from accessing routine prenatal care.

*“In Afghanistan, since the economic situation is so bad, people cannot afford to make an appointment with doctors more frequently the way they can here. Usually, you only go to the doctor when you are in severe trouble, or there is something urgent”* (Participant 3).

The participants described a lack of attention to personal health and heavy physical work in Afghanistan.

*“In Afghanistan it was hard to think about my own health; it was not a thing. I was like, ‘Okay, I’m pregnant.’ I didn’t even realize my blood sugar was elevated until I came here”* (Participant 1).

*“We lived in a joint family, so there was more workload on me. Now, I only need to take care of my kids, and that's easy for me while pregnant”* (Participant 8).

Another woman described her life in Afghanistan when pregnant:

*“We didn’t have water at home, so we would have to bring water from the river so that was physically hard. I had a lot of fieldwork, animals, and a farm. Here in the US, everything is provided, so it’s really good”* (Participant 20).

Most of the women described a “healthy pregnancy” as one that was void of pain or abnormal bleeding. They self-relied on eating healthy and avoiding lifting heavy things to have a healthy pregnancy, which they found easier to do in the US.

*“Back in Afghanistan, the fact that there’s a lot of poverty, so nutrition was a big difficulty during the pregnancy due to our poor economic status. Here, the nutrition is not a problem, so I could eat well”* (Participant 8).

#### Birthing

Most participants spoke positively about giving birth in the US. They felt that the nurses and physicians were kind and attentive. As 19 of the 20 women had previously given birth in Afghanistan, they shared some contrasts. They explained that there were not enough physicians or nurses in Afghanistan to care for women in labor. As a result, they gave birth at home or in a hospital room with several other women. They were not allowed to have family members with them, and there were not enough nurses to give them attention, so they were on their own.

*“[Giving birth in Afghanistan] was bad in a sense because in one room there are 20 to 30 women giving birth at the same time”* (Participant 9).

One participant described her experience of having an episiotomy during birth without any medications in Afghanistan. When she went home, her pain worsened. She was told it would heal in a week, but it did not. It became infected*.*

*“I was in pain and suffering for a month and a half because the surgical site was badly infected; I couldn’t take care of the baby. It was an extremely painful and miserable experience. It was like giving birth not only once but 3 or 4 times. It was a disaster”* (Participant 12).

The women appreciated the professional care they received from doctors and nurses in the US.

*“Then the nurses and doctors, when they found out that my husband was not there, they were trying to comfort me. It was a really good experience. I didn't even realize my husband was not there. I was really happy that they were so mindful and were there for me. They would make sure that I understand and explain the steps to me, like, ‘If things go wrong, then we will probably do a cesarean-section.’ They would tell me ahead of the time and make sure that I understand, and then explain the steps”* (Participant 3).

In contrast,

*“Back in Afghanistan, the doctors were aggressive sometimes. They didn't have respect. They would cuss around me while I was giving birth which was disrespectful”* (Participant 2).

In addition to appreciating the care and attention they received in the US, half of the women were grateful for the epidural (10 of the 20 participants).

*“During my prenatal visits, the doctor explained that the epidural is an option. The experience was pleasant because I didn’t have to feel any pain”* (Participant 13).

Two of the women explained common perceptions among Afghan women regarding epidurals were that the procedure caused chronic headaches, led to cesarean deliveries, and caused paralysis.

*“The majority of Afghan ladies want to go through the birthing process naturally. They don’t want to be induced. I got induced both times I gave birth in the US versus back home; it was all-natural, and it went smoothly compared with the US. The [US] experience was not pleasant for me, and I didn’t even know why I was being induced. Maybe it was because I was not exercising well?”* (Participant 10).

Two of the participants reported having cesarean delivery in the US. One participant was frustrated that she had to have a cesarean birth. She preferred natural births. The doctor was concerned that there was too little amniotic fluid, so they told her she needed a cesarean delivery. Afterward, the doctor admitted to her that it was not necessary.

*“I feel really violated; the fact that the doctors never listen to me, and then afterward admitted that there was no need for the cesarean birth. The fact that I don’t speak English properly and don’t have financial stability. That made me really frustrated. After that procedure, I am not back to normal; I still have aches and pains. It’s really devastating for me to not be able to take care of my kids”* (Participant 17).

The other issue brought up by six of the participants was how uncomfortable they felt when there were males in the birthing room. Some women did not even want their husbands in the delivery room.

*“I did not prefer to have my husband in the delivery room because I felt uncomfortable. My husband was not comfortable either, but felt it was expected”* (Participant 19).

Participant 11 told a story about a friend who went to the hospital in labor, and there was a male doctor.

*“When they saw there was only a male doctor, they packed up and went home to give birth. The poor lady gave birth, and then it got complicated by a severe hemorrhage, so they got nervous and called the ambulance”* (Participant 11).

Another participant went through birth without an interpreter because a male was the only interpreter available at the hospital.

*“I just told them I don’t want the interpreter because he is a man, and I cannot have him there while I’m having the baby”* (Participant 4).

#### Postpartum

The postpartum experiences regarding breastfeeding were overwhelmingly positive. All the women except for one explained that breastfeeding was a usual and customary practice and that they breastfed their children for 2 years.

*“I don’t have any problem with breastfeeding my children; it’s a really smooth process.”* (Participant 12).

Regarding child spacing, the women favored space between their children; their preferences ranged from 2 to 5 years between each child. The contraceptive methods they preferred varied. One mentioned she had a tubal ligation after her seventh child. A few of the women used the copper IUD, and others used condoms and oral contraceptives. Those who used the injection (Depo Provera) did not like the weight gain. Those with the implant perceived it to cause depression, headaches, and nausea. A few of the women who tried oral contraceptives stopped due to their side effects and resorted to the natural method.

*“I do not prefer medication. I just like the natural way to not have kids”* (Participant 15).

Some women discussed the lack of information provided by healthcare providers about contraceptive options.

“*A lot of these Afghan ladies want to have some contraceptive after birth. But the fact that they don’t get a follow-up with their obstetrician, and they’re not educated enough that they can get pregnant right after giving birth, so before they even go to the OB, they’re already pregnant again”* (Participant 5).

Another participant shared that they need additional contraceptive education.

*“It’s hard to take care of ourselves with these back-to-back children. We don’t know about the birth control options; we tend to stop them if we have any adverse effects”* (Participant 12).

### Communication

The women noted that the most difficult issue in the US was the language barrier, but also the discordant cultural values related to their role as a woman and the sensitivity of reproductive health topics.

Language barrier: As the women reflected on their maternal health experiences, they identified the most challenging concern to be the language barrier, which they did not face in Afghanistan.

*“The biggest problem among Afghan women here is the fact that they don't know the language, so it just makes it really difficult for them to do anything for themself”* (Participant 11).

Most of the women stated that their husbands or professional interpreters helped them understand the childbirth protocol in the US. Some women preferred their husbands to interpret rather than the professional interpreter.

*“We were given the option of an interpreter, but I preferred my husband to do the interpretation for me because that way, I understand it better”* (Participant 7).

Women described instances when only a male interpreter was available, so they opted to be quiet or have a family member interpret instead.

*“Then it gets difficult if the interpreter is a male. That’s when we are not comfortable sharing concerns, and we usually don’t talk about it. We keep it to ourselves ’cause we are not comfortable talking about any reproductive concerns”* (Participant 9).

One participant was frustrated with the interpreter.

*“I took my son with me to the appointment, and I was having some trouble. When I was there, the interpreter translated my message completely differently to the doctor, and my son noticed and told him, 'Hey, that's wrong. That's not what my mom is saying.' Then the interpreter got mad at the little kid, ‘How dare you tell me I'm wrong? I'm the interpreter. I know what I'm saying.’ Yeah, we had a big problem, not with the doctor or the staff, but the interpreter was not communicating the message right”* (Participant 2).

Most women acknowledged having access to interpretation services at the facility where they were treated, but at times, misunderstandings occurred.

*“When I went to give birth they asked me, ‘Are you having a boy or a girl?’ I didn't understand what boy or girl meant, so I told them, ‘I'm having a boy.’ They arranged paperwork for circumcision and all that, and then I couldn't understand so I signed everything. And then a nurse who spoke Farsi came and explained what boy and girl meant. And then I told them, ‘No. I'm having a girl,’ so they had to cancel the whole paperwork and all that”* (Participant 5).

Relationship with husband: Given that all the participants were married, their relationship with their husbands was a common topic that revealed aspects of their culture and affected their communication with healthcare providers. The women's narratives were intertwined with a strong sense of respect, reliance, and appreciation for their husbands, yet at the same time, a shared understanding of traditional Afghan culture of women submitting to men as the decision-makers. The participants described both satisfaction with more freedom for women in the US, but also fear and challenges with the discordant cultures.

When asked what actions women took concerning a health problem, most said they would share the problem with their husbands first. He would decide what to do next, such as making an appointment with the doctor.

*“If I’m not comfortable sharing my health concern with the doctor, then I share it with my husband, then he will talk to the doctor”* (Participant 14).

Other women shared how grateful they were for their husbands. Participant 7 described complicated pregnancies that included hyperemesis and diabetes. When asked how she managed, she responded,

*“My husband was the biggest support for me. He would try to look after our little girl and tried to help with the house chores; he helped me record the blood sugar levels and taught me how to operate the machine. My husband is supportive so that helped me through that pregnancy.”*

Some of the participants felt it would be helpful for healthcare providers to understand traditional marital dynamics for Afghan families. Since the husband makes the decisions for the wife, healthcare providers should provide health recommendations to the husband.

*“Ladies really respect their husbands. If the husband wants to have more kids, they don’t tell their husbands, ‘Hey, I cannot do it. My body is not able to get another pregnancy to term.’ Husbands don’t usually listen to their wives, but they do listen to the doctor if the doctor tells them, ‘Your wife needs some time before she gets pregnant again.’ They will most likely take that into consideration instead of their wives telling them”* (Participant 3).

Despite one participant sharing stories about other women dealing with a controlling husband, most of the women felt that their husbands were supportive and were attentive to their needs.

Women’s Information Seeking: The women shared that they usually only discuss reproductive issues with their close female family members. Many of them did not even learn about childbearing issues until they were pregnant themselves.

*“I learned about pregnancy and childbirth by seeing other family members going through the process and talking to females in the family”* (Participant 10).

Participant 14 commented,

*“No one ever really talked about it--like how pregnancy happened or what the nine months of pregnancy is like and then the delivery.”*

A couple of the women stated that by living in the US, they had learned to search the internet to educate themselves on their concerns or questions. For example, a participant wondered about having sex during pregnancy and was too shy to ask the doctor. She also wondered which sleeping position was best during pregnancy.

*“I researched Google and found that sleeping on the left side and using a pregnancy pillow is a good option”* (Participant 10).

The participants were in favor of educational sessions on reproductive health and contraceptive options, even though they described the cultural taboo surrounding discussing reproductive health.

*“It’s considered a shameful topic or taboo; it’s not a topic that women will share with each other or talk about it in a gathering, so usually they keep it to themselves”* (Participant 10).

Four of the participants explained that Afghan women are “shy.” They are afraid to talk about their reproductive health problems. A participant described the difference between women who came from more conservative rural areas and women who lived in the city.

*“When it comes to ladies from villages, they don’t know how to get help for themselves. Sometimes they miss their appointments and they’re shy. They are afraid to tell the doctor about it, such as problems with their period”* (Participant 3).

Participants appreciated anticipatory guidance and explanations of processes. Three women felt the doctor did not explain the medical interventions (Participants 5, 10, 17). Several women expressed interest in learning more about reproductive health and child spacing options. They felt that Afghan women needed educational sessions and language classes.

*“Yeah, so after my second daughter, when I went for the postpartum visit they said, ‘Oh, you're not having bleeding and everything. You're done with us and we're not going to be seeing you anymore because you gave birth, and everything looks okay.’ I was not even given any information; I didn't understand anything because I couldn't speak English. I was not given any information about contraceptives. I asked people from Afghanistan to send me birth control medication so I could use that as a contraceptive”* (Participant 5).

When probed further about preferences for health education sessions, Participant 8 commented,

“*A lot of Afghan ladies, they have kids up to seven and eight. Usually, they are not able to go anywhere—just because they have a lot of kids. If somebody came into our house and gave us information; that would be so helpful.”*

The other common concern was that the women were busy with childcare responsibilities that left no time for socializing with other women.

*“I stay very busy with my kids and don’t get together with other Afghans. I never have conversations to see what some of the problems are that they are facing or how I could help those problems”* (Participant 6).

Another participant wished women could get together but concurred,

*“The fact that most of these women have a lot of kids to take care of. They don’t drive—they are just homebound”* (Participant 9).

Access to Care: The women identified structural barriers they encountered in the US as they sought healthcare services.

Transportation: One of the primary barriers for women attending prenatal care appointments was the lack of transportation. Only one out of the twenty women mentioned that she could drive herself and had a car. The others relied on their husbands to drive them.

*“On the days my husband went to work, I would just cancel the appointment because I couldn’t drive”* (Participant 5).

Several women noted that this was a problem common to Afghan women.

*“The biggest challenge for women in the Afghan community is transportation. Most of us do not drive, so we cannot make the doctor’s appointments. We must wait for our husbands*” (Participant 17).

Financing Healthcare: Most women reported having Medicaid (government sponsored healthcare for low-income families) or some form of insurance coverage. However, some mentioned that when their pregnancy coverage ran out, they did not know how to navigate follow-up care.

*“If you have insurance, then you can go to the doctor; if not, then you are just on your own. One of the new Afghan refugees is nine months pregnant and doesn’t have insurance or any paperwork. She is frail. I tried to help, but she doesn’t have health insurance. I don’t know what to do”* (Participant 2).

The participants stated that the primary healthcare resource women need is Medicaid.

“*Some of these Afghan ladies have been sick for a long time. If the government can help, it would be helpful because most Afghan ladies stay home sick. They fear they will go to a doctor and be sent a high bill, which they cannot pay. It’s just so hard when you are sick and have many kids to look after”* (Participant 8).

Most women noted that their financial situations had improved since resettling in the US. However, some mentioned the financial strain on their husbands when making ends meet and their desire to assist with income generation.

*“The husband is the only one that brings income to the house. That’s used up monthly. Women are trying, but they’re unable to help their husbands with the income”* (Participant 8).

## Discussion

The findings from this study contribute critical insights into Afghan women's perceptions, preferences, and maternal health experiences in the US. We found that Afghan women appreciated the kind, mindful, and attentive care of the nurses and physicians providing their reproductive healthcare. They contrasted their US experiences with what they were used to in Afghanistan, describing extremely resource-poor conditions. Similarly, Shafiei et al. [26] found that Afghan women in Australia felt healthcare provider’s caring attitude was the most critical factor in their perception of maternity care. In contrast, Afghan refugee women in Iran attributed their poor maternal health experience to discrimination and a lack of attention. They felt that their concerns were ignored and that they were given incompetent midwives and doctors. These experiences caused mistrust and reluctance to utilize health services [20]. This may be that women who have poor maternal-child outcomes are more likely to have negative perceptions of the care provided. Most of the women in our study did not perceive discrimination. Those who described negative experiences with maternal care in the US were those who underwent medical interventions, including labor inductions, vacuum deliveries, and cesarean sections. Despite the participants’ preference for “natural births,” half of them were in favor of having an epidural during labor, especially if the procedure was explained ahead of time. The US has one of the highest cesarean delivery rates among countries in the Organization for Economic Co-operation and Development (32.1%) [27]. In contrast, the World Health Organization reported a cesarean delivery rate of 2.7% in Afghanistan [28]. The difference in maternity care practices can contribute to discordant expectations and disappointment, as was evident with a participant in our study who attributed her chronic pain to an unnecessary cesarean delivery. Disappointment often led to mistrust and reluctance to access healthcare services.

Our findings point to a connection between communication and informed decision-making. Communication was a crucial factor in our participants’ maternal health experiences. A study in California with older Afghan refugee women found that miscommunication led to mistrust of healthcare providers [29]. Similarly, a study with Syrian refugees in Norway found that poor communication resulted in more misunderstandings and negative doctor-patient interactions, which led to mistrust [30].

The language barrier affected their ability to express their feelings to the practitioner. Relying on the interpreter introduced various challenges as well. Compounding the language barrier, Afghan women face disadvantages related to health literacy that stem from exclusion from the education system in Afghanistan. A study in Afghanistan found a correlation between low maternal health literacy and poor pregnancy outcomes [31]. The fact that 65% of our participants had no formal education and only 25% could read and write in their primary language speaks to the gap in health literacy and the potential for misunderstandings and untoward outcomes. Researchers in Australia found that tailoring maternal health anticipatory guidance according to health literacy levels was essential for the quality care of Afghan women [16]. Health practitioners should consider using visual or audio rather than written material to improve communication and health literacy. A study in Afghanistan found a high satisfaction rate with community health workers who used a computer tablet-based health video library to enhance health counseling sessions [32].

Another clear cultural preference was for only female practitioners and interpreters. The women did not feel comfortable explaining their symptoms—especially those pertaining to the reproductive tract. Having a male in the room made it even more challenging to express their concerns. Some studies found Afghan women were embarrassed to ask questions because of their illiteracy [16, 26]. Our participants did not mention this factor but explained that reproductive health is a taboo topic and not even discussed among their female friends and social circles. They lacked the time and opportunity to get together socially because of childcare responsibilities. In a scoping review of refugee maternal health in the US, the authors found a need for healthcare provider cultural sensitivity and to include ethnic groups that are invisible in the published research [15]. Even though Muslim populations in the US share common values, our study is a step toward considering distinct cultural factors that affect the quality of care for Afghan women, including the importance of the husband's support and the impact of having a male in the room [33]. Healthcare providers in the US should strive for gender concordant healthcare professionals and interpreters given that women tend to avoid communicating if a male is present.

Cultural insights revealed a complex interplay in the husband-wife relationship marked by a common understanding that the husband was the decision-maker, and the women primarily relied on their husbands for interpretation, transportation, and communicating with healthcare providers. They expressed gratefulness when their husband assisted and supported them through the process of pregnancy and birthing. They reflected that in Afghanistan, female family members provided support during pregnancy and childbirth; so, in the US, where they lacked extended family, they relied more on their husbands.

This group of 20 women had been in the US for an average of 5 years; therefore, their views and preferences had likely evolved as they assimilated into the US. None of the women mentioned experiencing violence from their husbands. Yet, surveys within Afghanistan from 2015 revealed that 46.1% of women experienced sexual or physical violence from their husbands [34]. Our study and other studies of Afghan refugee women revealed complementary husband-wife bonds through childbearing [35]. Afghan women in Iran talked about how their husbands gave them sacrificial support—even begging for money in the streets to pay hospital bills [19]. Given our findings, US healthcare providers must avoid making assumptions about the husband-wife relationship; instead, they should respect and nurture the strengths it brings and consider including them in reproductive health conversations.

All the women, except one, reported breastfeeding their children for 2 years. As 100% of the women identified as Muslim, it is worth noting that the Quran states that a child has a right to receive breastmilk until the age of 2 years [36]. Because the health benefits of breastfeeding extend to both mother and baby, healthcare providers and systems should encourage sustainment of this practice throughout the assimilation process and subsequent generations.

Participants were open to using child spacing methods. They reported using a variety of contraception types. None of the women mentioned religious opposition to contraception but mentioned adverse effects that drove them to natural contraceptive methods. The findings from studies in Australia also cited that both men and women were open to contraception, but the preferences varied due to side effects [37]. Even studies in refugee camps in Asia found a rate of 54% contraception utilization among Afghan women [38]. Aside from the challenges with contraceptive side effects, our findings revealed a lack of communication between the provider and the woman about child spacing options and indicated a need for improved health education related to contraception.

The women outlined several barriers to accessing healthcare. They first needed approval from their husbands, then transportation and language interpretation assistance. If they did not have Medicaid coverage, they were unlikely to seek care unless it was a life-or-death situation. Studies in Australia found similar dynamics, with Afghan men playing significant roles in supporting their wives throughout the pregnancy and birth process. Health professionals rarely engaged with the participants’ husbands regarding their concerns or perspectives on the pregnancy [35]. Within the US culture of emphasizing patient autonomy, health professionals need to reinvigorate their approach to Afghan families and engage husbands to accentuate the strengths of their support while considering the social and economic burdens Afghan men face. Alternatively, given the multiple demands on husbands and cultural tensions of having husbands involved in the birth process, further research into the acceptability of having female doulas provide support for Afghan women should be explored. Doula support has been shown to improve birth outcomes, including reduced cesarean sections, use of instrumental vaginal birth, less analgesia, shortened labor time, and fewer infants with low Apgar scores [39].

### Strengths and limitations

A strength of this study was that one of the authors from Afghanistan was fluent in all three languages. Having an all-female research team, including the interpreter, broke the language and gender barriers. The interpreter is also a physician with the medical background to understand the terminology and processes discussed by and with the women. These factors likely contributed to the woman’s comfort level in sharing their experiences related to this culturally taboo topic.

A limitation of this study is that it was a convenience sample. The women who agreed to participate may have different perceptions than women who declined the interview. For example, some of our participants seemed to take on the role of advocating for another woman. They shared examples of how they tried to encourage women to speak up and share their concerns with the doctor rather than keep it to themselves. Interpretation of the results should keep these factors in mind. Another limitation was that the interviews were conducted in various venues depending on the participant’s preference, so some of the interviews were in-person, but the majority were over the phone. Lack of body language and background noise over the telephone may have affected the clarity of what the participant said.

## Conclusions

The fact that the Afghan culture is strikingly different than the US mainstream culture can lead to stereotypical assumptions, poor communication, and poor health outcomes.

The voices of Afghan women should guide healthcare providers in delivering patient-centered, culturally sensitive maternity care that promotes healthy families and communities. We found attitudes of gratefulness, strong husband support, commitment to breastfeeding, openness to child spacing, and desire to learn more about reproductive health. Interventions should address communication barriers, discordant cultural values, the lack of social/community networks, and the lack of transportation.

### Supplementary Information

Supplementary Material 1.

## Data Availability

The fully anonymized datasets used and/or analyzed during the current study are available from the corresponding author on reasonable request from authenticated researchers.

## Abbreviation

US: United States
