# Supplementary material for: “It’s different here” Afghan refugee maternal health experiences in the United States
Source: BMC Pregnancy Childbirth. 2024 Jul 16;24:479. doi: 10.1186/s12884-024-06678-7 (PMC11251342; doi:10.1186/s12884-024-06678-7)
Supplement: Supplementary file 1 — Supplementary Material 1. [file 12884_2024_6678_MOESM1_ESM.docx]

**Appendix A –** Demographic Form

Age:

Marital status:

Number of pregnancies:

Number of children:

Number of children born in the US:

Length of time in the U.S.:

Primary language:

English proficiency: (none, conversational, fluent)

Country of origin:

Religion:

Employment: (yes/no)

Health insurance: (yes/no)

Primary care provider: (yes/no)

Education: (none, elementary, secondary, college)

**Appendix B -** Initial Interview Guide with Participants 1-8

1. Tell me about your pregnancy experience living in the United States.

a. What was good?

b. What wasn’t good?

c. How was being pregnant in the U.S. different from being pregnant in your home country (if prior birth in your home country)?

2. Tell me about your birth experience in the United States.

a. What was good?

b. What wasn’t good?

c. How was it different from giving birth in your home country (if prior birth in your home country)?

3. Tell me about the most important health issues for childbearing women in your community.

a. What healthcare resources do women like you need to take care of yourselves before, during, and after childbirth?

**Appendix C -** Revised Interview Guide for Participants 9-20

1. Tell me about your pregnancy experience while living in the United States.

a. What was good?

b. What wasn’t good?

c. How was being pregnant in the U.S. different from being pregnant in your home country (if prior birth in your home country)?

*d. What questions do you have about pregnancy? What are your concerns? During your pregnancy, how do you know that everything is okay with the baby? How do you know when something isn’t ok? Did you worry about anything during pregnancy?*

*e. What are your beliefs about what it is like to have a healthy pregnancy? How do you take care of yourself during pregnancy?*

2. Tell me about your birthing experience in the United States.

a. What was good?

b. What wasn’t good?

c. How was it different from giving birth in your home country (if prior birth in your home country)?

*d. How did you learn about pregnancy and childbirth? Who do you learn from?*

*e. What was your experience with accessing healthcare in between your pregnancies (i.e., postpartum visit at 6 weeks)?*

*f. What are your preferences with child spacing?*

*g. Do you have any concerns about breastfeeding?*

3. Tell me about the most important health issues for childbearing women in your community.

a. What healthcare resources do women like you need to take care of yourselves before, during, and after childbirth?

*b. What help do pregnant women and mothers need?*

*c. If you have a question about your health, who do you ask?*

*d. What things do women share with each other about pregnancy and birth?*

*e. What kind of problems do women go to the doctor for? Are there concerns that you don’t want to share with the doctor? Who would you share with?*
